# Speciation by genome duplication: Repeated origins and genomic composition of the recently formed allopolyploid species *Mimulus peregrinus*

**DOI:** 10.1111/evo.12678

**Published:** 2015-05-27

**Authors:** Mario Vallejo‐Marín, Richard J. A. Buggs, Arielle M. Cooley, Joshua R. Puzey

**Affiliations:** ^1^ Biological and Environmental SciencesUniversity of StirlingStirlingFK9 4LAUnited Kingdom; ^2^ School of Biological and Chemical SciencesQueen Mary University of LondonLondonE1 4NSUnited Kingdom; ^3^ Biology DepartmentWhitman CollegeWalla WallaWashington99362; ^4^ Department of BiologyCollege of William and MaryWilliamsburgVirginia23185

**Keywords:** Hybridization, invasive species, plant speciation, polyploidy, whole genome duplication

## Abstract

Whole genome duplication (polyploidization) is a mechanism of “instantaneous” species formation that has played a major role in the evolutionary history of plants. Much of what we know about the early evolution of polyploids is based upon studies of a handful of recently formed species. A new polyploid hybrid (allopolyploid) species *Mimulus peregrinus*, formed within the last 140 years, was recently discovered on the Scottish mainland and corroborated by chromosome counts. Here, using targeted, high‐depth sequencing of 1200 genic regions, we confirm the parental origins of this new species from *M. x robertsii*, a sterile triploid hybrid between the two introduced species *M. guttatus* and *M. luteus* that are naturalized and widespread in the United Kingdom. We also report a new population of *M. peregrinus* on the Orkney Islands and demonstrate that populations on the Scottish mainland and Orkney Islands arose independently via genome duplication from local populations of *M. x robertsii*. Our data raise the possibility that some alleles are already being lost in the evolving *M. peregrinus* genomes. The recent origins of a new species of the ecological model genus *Mimulus* via allopolyploidization provide a powerful opportunity to explore the early stages of hybridization and genome duplication in naturally evolved lineages.

Whole genome duplication, or polyploidization, has been linked to major evolutionary transitions in the history of land plants including the diversification of angiosperms (Levin 1983; Cui et al. 2006; Soltis et al. 2014). Approximately 35% of angiosperm species are recent polyploids, and 15% of speciation events in angiosperms are directly associated with genome duplication (Wood et al. 2009). Polyploidization is often correlated with hybridization events (Abbott et al. 2013), and the combination of hybridization and polyploidy (allopolyploidy) is thought to contribute to the rapid evolution of genome structure and ecological novelty (Song et al. 1995; Wright et al. 1998; Osborn et al. 2003; Buggs et al. 2012a; Chao et al. 2013; Soltis et al. 2014). However, our ability to study the contribution of hybridization and polyploidy to evolutionary change in established allopolyploids is limited as it is not easy to distinguish the changes happening at the moment of hybridization and polyploidization from those accumulating after subsequent evolution. Recently formed allopolyploid species provide opportunities to investigate the earliest stages of speciation by genome duplication. Examples of recently formed allopolyploid species (< 200 years old) include only a handful of species in four families: *Senecio cambrensis, S. eboracensis* (Abbott and Lowe 2004), *Tragopogon mirus* and *T. miscellus* (Soltis et al. 2004) in the Asteraceae, *Cardamine x insueta* and *C. x schulzii* in the Brassicaceae (Mandakova et al. 2013), *Spartina anglica* in the Poaceae (Ainouche et al. 2004), and *Salsola ryanii* in the Amaranthaceae (Ayres et al. 2009). Generalizations of the evolutionary changes observed during polyploid speciation clearly will benefit from the study of additional young allopolyploids.

The recent discovery of an allopolyploid species of monkeyflower, *Mimulus peregrinus* (Phrymaceae), formed in the last 140 years provides an exciting opportunity to study the early stages of speciation by genome duplication (Vallejo‐Marín 2012). The monkeyflower genus, *Mimulus L*. (Phrymaceae), comprises approximately 120 species of herbaceous plants with centers of diversity in North America where the majority of species occur, and also the Andes, the Himalayas, and Australia (Grant 1924; Beardsley and Olmstead 2002; Wu et al. 2007). *Mimulus* has served as a model to study ecology, evolution, and speciation in plants for more than 50 years (Vickery 1959; Bradshaw and Schemske 2003; Wu et al. 2007; Lowry and Willis 2010), but only recently have studies begun investigating the ecological and evolutionary dynamics of *Mimulus* species in their nonnative range (van Kleunen 2007; Murren et al. 2009; Puzey and Vallejo‐Marín 2014).

Monkeyflowers were introduced into Europe from the Americas in the early 1800s, and detailed accounts of the history of colonization of this genus in Europe are provided elsewhere (Tokarska‐Guzik and Dajdok 2010; Vallejo‐Marín 2012; Puzey and Vallejo‐Marín 2014). The two most successful colonization events involved the North American taxon *Mimulus guttatus*, which was introduced into Europe in 1812, closely followed by taxa in the South American species‐aggregate *M. luteus* (Vallejo‐Marín and Lye 2013). In their native range, *M. guttatus* has a variable mating system from high to intermediate levels of outcrossing (e.g., Willis 1993), and *M. luteus* is also likely to be partially to predominantly outcrossed (Cooley et al. 2008). In the United Kingdom, *M. guttatus* is naturalized and widespread along the banks of streams and rivers, in water‐logged ground and other wet places (Preston et al. 2002; Truscott et al. 2008). *Mimulus luteus* inhabits similar environments, and also became naturalized and widespread soon after its introduction to the United Kingdom (BSBI 2013). However, *M. luteus* is currently much rarer than *M. guttatus* (Vallejo‐Marín and Lye 2013). The mating system of introduced *Mimulus* is unknown. *Mimulus guttatus* and *M. luteus* are closely related, belonging to the section *Simiolus* (Grant 1924), but, importantly, differ in ploidy level: while *M. guttatus* occurs mostly as a diploid (2*n* = 2x = 28), taxa in the *M. luteus* group are tetraploid (2*n* = 4x = 60–62; Vickery 1995). In the United Kingdom, *M. guttatus* and *M. luteus* hybridize to produce a sexually sterile, but vegetatively vigorous triploid taxon, *M. x robertsii* (2*n* = 3x = 44–46; Roberts 1964; Parker 1975; Silverside 1990). This sterile hybrid is found in approximately 40% of extant *Mimulus* populations throughout the United Kingdom, and can form very large populations of thousands of ramets thanks to its ability to reproduce from even small plant fragments (Vallejo‐Marín and Lye 2013).

*Mimulus peregrinus* is a newly described taxon recently found in mainland Scotland. It shares the morphological characteristics of the *M. x robertsii* triploid hybrids, but possesses twice the genome size and number of chromosomes (2*n* = 6x = 92), and is characterized by high (>90%) pollen fertility (Vallejo‐Marín 2012). Based on these phenotypic traits, and the fact that triploid hybrids have long been known to give rise to fertile species via whole genome duplication (e.g., Ainouche et al. 2004; Hegarty et al. 2012), it appears that *M. peregrinus* has arisen through allopolyploidization between introduced populations of *M. guttatus* and *M. luteus* (Vallejo‐Marín 2012). In addition to the originally described (Vallejo‐Marín 2012) population of *M. peregrinus* in mainland Scotland (Leadhills, South Lanarkshire), polyploid individuals of similar morphology have been found in a separate stream nearby (5 km away), and also 400 km to the north of the type location, on the Orkney Islands, which are separated from the Scottish mainland by 16 km of ocean. Populations of *M. x robertsii* are also present on Orkney. Given molecular evidence suggesting that multiple origins of several neoallopolyploid species (Soltis and Soltis 1999; Soltis et al. 2009) such as *S. cambrensis* in the United Kingdom (Ashton and Abbott 1992), and *Tragopogon miscellus* and *T. mirus* in Idaho and Washington (Symonds et al. 2010), we hypothesized that *M. peregrinus* populations on mainland Scotland and Orkney had independent origins. A genome‐level study has the potential to rigorously test these hypotheses on the identity and repeated origins of *M. peregrinus*.

Here, we use high‐depth targeted sequencing of 1200 genic regions to characterize the genomic composition of *M. peregrinus* individuals from two populations in Southern Scotland and the newly discovered population in the Orkney Islands. We compare these with the same regions sequenced at high coverage in the parental taxa, *M. guttatus* and *M. luteus s. l.*, and in *the M. x robertsii* triploid hybrids. We address two specific questions: (1) Is the genome composition of *M. peregrinus* consistent with recent allopolyploidization from *M. guttatus* and *M. luteus* via *M. x robertsii*? (2) Has *M. peregrinus* originated multiple times?

## Materials and Methods

### SAMPLING

**Figure 1:**
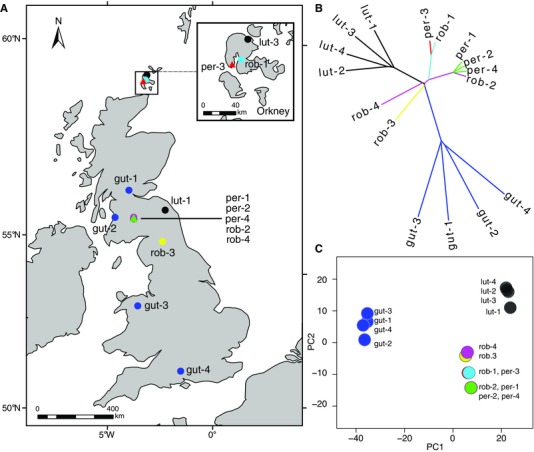
Geographic location and genomic relationships between 16 individuals of *Mimulus* spp. in each of four taxa: *M. guttatus* (gut), *M. luteus* (lut), *M. x robertsii* (rob), and *M. peregrinus* (per). (A) Map of the United Kingdom showing the geographic location of the samples analyzed here. Two individuals of *M. luteus* (lut‐2 and lut‐4) were collected in Chile and are not shown in this map. (B) Neighbor joining tree showing monophyletic clades for the parental taxa (*M. guttatus* and *M. luteus*), and clustering of the allopolyploid *M. peregrinus* with geographically proximate populations of *M. x robertsii*. Bootstrap support for all nodes is shown in Figure S3. (C) Principal component analysis (PCA) plot showing clustering of individuals along the first two principle components. *Mimulus peregrinus* and *M. robertsii* fall between parental species, *M. guttatus* and *M. luteus* and next to geographically proximate *M. x robertsii*.

To elucidate the relationships between *M. peregrinus* and its putative parental taxa, we sampled four individuals of each of *M. guttatus*, *M. luteus*, *M. x robertsii*, and *M. peregrinus*, identified on the basis of morphology and genome size measured using flow cytometry (Table 1, Fig. 1A). For *M. guttatus*, we sampled four individuals from natural populations in the United Kingdom. For *M. luteus*, we included the only two extant naturalized populations of this taxon that we could find in the United Kingdom, plus two advanced generation inbred lines, *M. luteus var. luteus* and *M. luteus var. variegatus*, both of which are field‐collected varieties originating from Chile (Cooley and Willis 2009), and hypothesized to have contributed to naturalized populations of *M. luteus* in the United Kingdom (Stace 2010; Vallejo‐Marín 2012). Other related taxa such as *M. naiandinus* and *M. cupreus* are not naturalized in the United Kingdom (Stace 2010). For *M. peregrinus*, we obtained samples from two populations LED (sampled twice, Table 1) and GON occurring in close proximity to each other (∼5 km) but on different streams in South Lanarkshire, and another population (STR) found approximately 400 km to the North, near Stromness on the Orkney Islands. Both populations, LED and GON, also contain individuals of *M. x robertsii* which we sampled. The STR population is composed only of *M. peregrinus* so we sampled the nearest *M. x robertsii* population, 7 km distant, near Maeshowe. We sampled a further *M. x robertsii* population from Nenthall, England, which is far from all known *M. peregrinus* populations.

**Table 1:** Summary characteristics of 16 individuals from four *Mimulus* taxa sampled for the targeted genome re‐sequencing analysis

					Expected			
					genomic	Percentage	Mean	Fraction
Sample ID	Population	Location	Latitude	Longitude	composition	of mapped	depth	heterozygous
*M. guttatus* (2x)								
gut‐1	DBL	Dunblane, Scotland	56.187	−3.965	GG	94.5	65	0.018
gut‐2	AYR	Ayr, Scotland	55.461	−4.625	GG	94.3	74	0.019
gut‐3	CER	Cerrigydrudion, Wales	53.006	−3.549	GG	94	83	0.021
gut‐4	HOU	Houghton Lodge, England	51.097	−1.508	GG	93.8	111	0.018
*M. luteus* (4x)								
lut‐1	COL	Coldstream, Scotland	55.655	−2.240	LLLL	88.2	90	0.047
lut‐2	EY	El Yeso, Chile	−33.4	−70.0	LLLL	87.9	96	0.044
lut‐3	EVI	Evie, Orkney, Scotland	59.112	−3.108	LLLL	88.6	74	0.051
lut‐4	RC	Río Cipreses, Chile	−34.2	−70.3	LLLL	87.5	90	0.045
*M. x robertsii* (3x)								
rob‐1	TOR	Tormiston, Orkney, Scotland	58.996	−3.183	GLL	89.3	112	0.067
rob‐2	LED	Leadhills, Scotland	55.424	−3.735	GLL	90.1	98	0.065
rob‐3	NEN	Nenthall, England	54.806	−2.376	GLL	89.8	106	0.066
rob‐4	GON	Glen Gonnar, Scotland	55.467	−3.738	GLL	89.7	102	0.066
*M. peregrinus* (6x)								
per‐1	LED	Leadhills, Scotland	55.423	−3.735	GGLLLL	88.9	90	0.064
per‐2	LED	Leadhills, Scotland	55.424	−3.735	GGLLLL	89.8	117	0.067
per‐3	STR	Stromness, Orkney, Scotland	58.969	−3.283	GGLLLL	90.8	82	0.064
per‐4	GON	Glen Gonnar, Scotland	55.467	3.738	GGLLLL	90.3	109	0.067

Taxon names are followed by estimates of ploidy level obtained using flow cytometry and/or chromosome counts. For chromosome counts and details on taxon identification, see Vallejo‐Marín (2012). *Latitude* and *Longitude* are expressed in decimal degrees in the WGS84 coordinate system. *Expected genomic composition* indicates the number of haploid complements expected from each of the putative parental taxa: *M. guttatus* (G) and *M. luteus* (L). Percentage of mapped: percent of total number reads mapped; mean depth: average read depth of genotyped sites; fraction heterozygous: fraction of heterozygous sites. Values for number sites, mean depth, and fraction het, were calculated from sites genotyped in all 16 individuals when running GATK in polyploidy mode allowing up to four alternate alleles.

### SELECTION AND SEQUENCING OF SEQUENCE CAPTURE BAITS

To carry out targeted genomic sequencing, we designed probes using the *M. guttatus* genome version 1.1 (www.phytozome.net, based on a North American accession of *M. guttatus*). We randomly chose coding genic and untranslated regions in a ratio of 3:1 for a total of 1198 regions of 450 bp each (540 kbp total) across the whole genome. For these target regions, we designed Agilent SureSelect RNA baits using the SureDesign software (Agilent Sure Select XT Enrichment Protocol version 1.6; Agilent Technologies, Stockport, Cheshire, UK). The final design yielded 20,385 probes with a total size of 488,076 bp. We extracted DNA from dried leaf tissue using DNeasy Plant Mini Kits (Qiagen, Crawley, West Sussex, UK) and used 1.5–7.9 μg of DNA for hybridization with the SureSelect RNA baits. The captured DNA was sequenced in a 100 bp pair‐end run in a MiSeq desktop sequencer (Illumina, Little Chesterford, Essex, UK) at the NERC Biomolecular Analysis Facility‐Edinburgh (Genepool, Edinburgh, UK). Fastq files were obtained using the Illumina pipeline CASAVA version 1.8.3.

### ANALYSES

#### Read alignment and genotyping

We aligned raw reads to the newest available version of the *M. guttatus* genome (version 2.0; www.phytozome.net) using *bowtie2*. Following initial alignment and conversion to binary format (*SAMtools*, Li et al. 2009), we used *Picard‐tools* (http://broadinstitute.github.io/picard) to validate mates, sort alignments, remove duplicates, and add read groups. After *Picard‐tool* filtering, we used the *Genome Analysis Toolkit Unified Genotyper* to call genotyping with base quality (BQ ≥ 25) and mapping quality (MQ ≥ 25) filters in place. After genotyping, we discarded indels and bases with genotype quality scores less than 30. Sites with two or more alternate alleles (as determined using GATK in polyploidy mode with four alternate alleles allowed) were discarded. Detailed commands and pipelines are provided in the Supplementary Information.

To map *M. guttatus* probes (designed with version 1 of the genome) to version 2 locations, we used *blastn* (NCBI C++ Toolkit). From all designed probes, 64% (772/1198) mapped uniquely to the version 2 genome, 7.7% probes (92/1198) had two hits with identity greater than 90%, and 13% (156/1198) mapped twice or more throughout the genome with identity greater than 72%. Figure S1 provides a genome‐wide plot showing the location of the probes. Only probes with one unique and perfect hit in the version 2 genome were used for subsequent analyses.

#### Genomic relationships between individuals

To reconstruct the relationships among the 16 sampled individuals, variable loci were selected from the sequenced reads. Heterozygotes were defined as those sites in which the frequency of the reference allele was between 0.1 and 0.90. Only SNPs (Single Nucleotide Polymorphisms) with a read depth of at least 50×, and which were amplified in all 16 individuals and polymorphic in at least one individual were retained for this analysis. Our genotype calling did not distinguish between different allele dosage types of heterozygotes in polyploid individuals (e.g., *guttatus*‐*guttatus*‐*luteus* vs. *guttatus*‐*luteus*‐*luteus*), but enabled us to directly compare presence/absence of alleles across taxa.

We used allele presence/absence calls at 20,749 SNP loci that were biallelic within our samples to calculate a pairwise Nei's genetic distance matrix among all individuals in the software *adegenet* (Jombart and Ahmed 2011) in *R* (R Development Core Team 2014), and used these data to generate a neighbor joining (NJ) tree in *ape* (Paradis et al. 2004). We calculated bootstrap values for the nodes in the NJ tree using 1000 replicates. For comparison, we also used these calls to conduct a principal component analysis (PCA) using the function *glPca* in *adegenet*. As PCA is a simple method to reduce dimensionality of multivariate data, it does not assume a population genetic or inheritance model, and collapses correlated information in linked SNP loci, providing a comparison for our NJ analysis with few assumptions.

#### Estimating allele dosage using loci with reciprocally fixed alleles in M. guttatus and M. luteus

We identified loci that are fixed for alternative alleles in *M. guttatus* and *M. luteus* using biallelic SNPs with a read depth of at least 50× sequenced in all 16 individuals. Reads from *M. x robertsii* and *M. peregrinus* that contain these SNPs can therefore be characterized according to which taxon they were derived from. For each locus/individual combination in *M. x robertsii* and *M. peregrinus*, we calculated the ratio of “*guttatus*” alleles to “*luteus*” alleles by counting reads for each allele at each locus. To ascertain whether these results might be influenced by hybridization bias by the probes for sequences from the reference genome that the probes were designed from (a North American *M. guttatus*), we analyzed separately the SNP loci in which our “*luteus*” allele is the same as the allele in the reference genome (and consequently our “*guttatus*” allele is the variant relative to the reference genome), and those loci in which our “*luteus*” allele is different from the allele in the reference genome (and hence our *guttatus* allele is the same as the reference genome).

#### Allelic variants unique to M. x robertsii or M. peregrinus

We searched for alleles that were unique to either *M. x robertsii* or *M. peregrinus*, that is, not present in either *M. guttatus* or *M. luteus*. To do this, we focused on biallelic SNP loci with a read depth of at least 50×, and which were sequenced in all 16 individuals. We looked for loci in which a given allele was observed in any individual of *M. x robertsii* or *M. peregrinus* at a frequency of at least *q* > 0.167 (the frequency of one out of six allele copies in a hexaploid), but which was not detected in any individual of *M. guttatus* and *M. luteus* (i.e., the frequency of this allele was less than *q* < 0.1 in the putative parental taxa).

#### Allelic variants differentiating M. peregrinus from M. x robertsii

We then searched for SNP alleles that are found in *M. peregrinus* but not in *M. x robertsii*, again focusing on biallelic SNP loci with a read depth of at least 50×, and sequenced in all 16 individuals. Among these, we selected sites that were fixed in all individuals of *M. x robertsii* (*q* > 0.9), and in which reads containing the alternative allele were found in any individual of *M. peregrinus* at a frequency of at least 1 − *q* ≥ 0.167 (i.e., equal or higher than the frequency of one out of six allele copies in a hexaploid). Finally, we looked for alleles that are found in *M. x robertsii* but not *M. peregrinus*. We searched for heterozygote sites (0.167 < *q* < 0.833) in *M. x robertsii*, and selected those that occurred as homozygotes in *M. peregrinus* using a slightly more conservative threshold (0.1 > *q* > 0.9).

## Results

The average sequencing depth at probed regions ranged from 113 to 202× per individual, with an overall average of 164× coverage (Table 1). Individuals from *M. guttatus* had slightly more aligned reads (94%) than other taxa (*M. luteus*: 88%, *M. robertsii*: 90%, and *M. peregrinus*: 90%; Table 1). On a per individual basis, the number of sites within probes confidently genotyped ranged from 338166 to 347182 with an average of 334419 sites (Table 1).

### GENOME‐WIDE HETEROZYGOSITY

Observed levels of genome‐wide heterozygosity varied extensively among the four taxa as indicated by the fraction of heterozygous sites (number of heterozygote sites/total sites genotyped; Table 1), and illustrated in Figure S2 using a subset of 20,749 SNPs with read depth >50× and genotyped in all individuals. Individuals of *M. guttatus* collected in naturalized populations in the United Kingdom had the lowest fraction of heterozygous sites, ranging from 0.018 to 0.021 (mean = 0.019 ± 0.001; mean ± SD; Table 1). This level of heterozygosity is consistent with a previous study of 10 U.K. populations of *M. guttatus*, which showed that the overall genome‐wide pairwise nucleotide diversity was π = 0.015 (Puzey and Vallejo‐Marín 2014). In contrast, individuals of the tetraploid *M. luteus* had over twice as many heterozygous bases (mean = 0.047 ± 0.003). This high level of heterozygosity was observed in both wild‐collected individuals of *M. luteus* in the United Kingdom (lut‐1 and lut‐3; fraction heterozygous sites = 0.047 and 0.051, respectively), as well as in two individuals from fifth generation inbred lines: *M. luteus var. luteus* (lut‐2; fraction heterozygous sites = 0.044) and *M. luteus var. variegatus* (lut‐4; fraction heterozygous sites = 0.0458; Table 1; Fig. S2). However, the highest levels of heterozygosity were observed in individuals of *M. x robertsii* and *M. peregrinus* (0.066 ± 0.001 and 0.065 ± 0.002, respectively; Table 1; Fig. S2), as would be expected if they are derived from a recent hybridization event between two distinct taxa.

### RELATIONSHIPS BETWEEN TAXA

The NJ analysis of the SNP data showed that *M. guttatus* and *M. luteus* form monophyletic clades clearly separated by relatively deep branches with very strong bootstrap support (Figs. 1B, S3). This analysis also supports a close association between *M. x robertsii* and *M. peregrinus*. The three individuals of *M. peregrinus* sampled from Southern Scotland (per‐1, per‐2, and per‐4; LED and GON populations) are most closely associated with *M. x robertsii* from the same geographic area (rob‐2; LED; Fig. 1B). Interestingly, although per‐4 (GON) co‐occurs with *M. x robertsii* (rob‐4; GON), it is actually more closely related to individuals from the Leadhills population located 5 km away (LED; per‐1, per‐2, and rob‐2; 100% bootstrap support for this node; Figs. 1B, S3). Furthermore, the *M. peregrinus* individual sampled in the Orkney Islands (per‐3; STR population) is most closely related to *M. x robertsii* from the same island (rob‐1; TOR population; Table 1, Fig. 1B).

The PCA grouped the populations in a manner similar to the relationships suggested by the NJ tree (Fig. 1C). The first principal component (PC) separated *M. guttatus, M. luteus*, and the hybrids (*M. x robertsii* and *M. peregrinus*), whereas the second PC provided some resolution within taxa. Importantly, the PCA shows that *M. robertsii* and *M. peregrinus* fall between *M. guttatus* and *M. luteus*, and indicates that *M. peregrinus* from each of the two main regions in Southern Scotland and Orkney are most closely associated to geographically proximate *M. x robertsii* (Fig. 1C).

### **GENOMIC COMPOSITION OF *M. X ROBERTSII* AND *M. PEREGRINUS***

**Figure 2:**
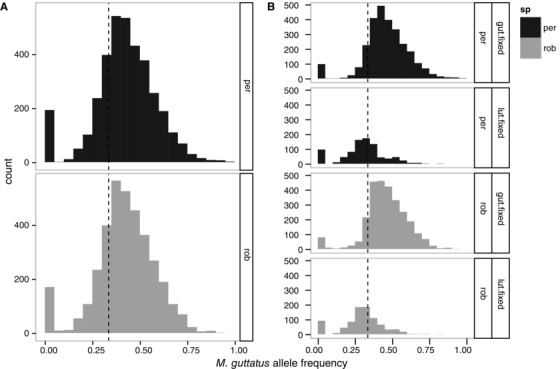
Histograms showing combined read depth frequency (allelic dosage) in four individuals of *M. peregrinus* (per) and four of *M. x robertsii* (rob) for SNP alleles that are fixed for alternative variants in the parental taxa *M. guttatus* and *M. luteus*. Only biallelic SNPs with a read depth of >50× and genotyped in all individuals were included in this analysis. The allelic frequency depicted in the x‐axis is for the allele coming from the diploid parent (*M. guttatus*). The dotted line indicates 1:2 ratio, expected in a hybrid product of the combination of diploid and tetraploid parental genomes. (A) All loci that are alternatively fixed in *M. guttatus* and *M. luteus*. (B) The same data but separated by whether SNPs alleles from *M. guttatus* are fixed for the reference allele and *M. luteus* are fixed for the alternate allele (gut‐fixed); or whether SNP alleles are fixed in *M. luteus* for the reference allele and *M. guttatus* is fixed for the alternate allele (lut‐fixed). Histogram bin size = 0.1.

In our sample of 20,749 SNP loci sequenced in all individuals, 93.78% loci only contained alleles in *M. x robertsii* and *M. peregrinus* that were also observed in *M. guttatus* and/or *M. luteus*, consistent with the hypothesis that *M. guttatus* and *M. luteus* are the parental species. To explore the contributions of these two species to the genomes of *M. x robertsii* and *M. peregrinus*, we identified 881 sites that were fixed for alternative alleles in all individuals of *M. guttatus* and *M. luteus* analyzed. These 881 informative sites were distributed in 370 different probes and included all 14 major linkage groups of the reference genome (Fig. S4). Figure 2 shows the distribution of read depth across these SNP loci across all individuals of *M. x robertsii* and *M. peregrinus*. The average frequency of the *M. guttatus* alleles estimated at each SNP loci separately was 0.418 ± 0.155 (mean ± SD) in *M. x robertsii*, and 0.420 ± 0.161 in *M. peregrinus*. The average frequency of the *M. guttatus* alleles when calculated as the mean frequency in each probe was 0.415 ± 0.076 and 0.413 ± 0.074 in *M. x robertsii* and *M. peregrinus*, respectively. If the tetraploid species *M. luteus* contributed twice as many genomes as the diploid *M. guttatus* to *M. x robertsii*, and *M. peregrinus*, the expected frequency of *M. guttatus* alleles in both *M. x robertsii* and *M. peregrinus* is 1/3. In this case, our results seem to indicate a slight overall deficit of *luteus*‐like reads (or an excess of *guttatus*‐like reads; Fig 2A).

To test whether a hybridization bias by our probes might be contributing to the apparent deficit of *luteus*‐like alleles in *M. x robertsii* and *M. peregrinus*, we analyzed separately the SNP loci in which the “*guttatus*” allele was the same as the allele in the reference genome and those loci in which the “*guttatus*” allele was different from the allele in the reference genome (Fig. 2B). We found that for loci in which the “*guttatus*” allele did not match the reference genome (*n* = 212 SNP loci in 118 probes), the allelic frequency of the *guttatus*‐like allele was 0.295 ± 0.136 and 0.300 ± 0.146 in *M. x robertsii* and *M. peregrinus*, respectively. In contrast, for loci in which the “*guttatus*” allele matched the reference genome (*n* = 669 SNP loci in 317 probes), the allelic frequency of the *guttatus*‐like allele was 0.458 ± 0.140 and 0.458 ± 0.147 for *M. x robertsii* and *M. peregrinus*, respectively. This finding suggests that there was a hybridization bias in our probes for alleles matching our reference genome; in general this inflated the observed frequency of *guttatus*‐like reads (Fig. 2).

**Figure 3:**
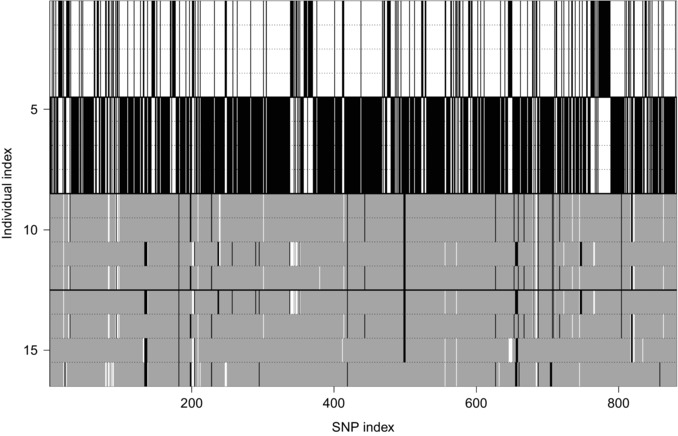
Genotype plot of 881 biallelic SNPs that are fixed for alternate alleles in *M. guttatus* and *M. luteus*. Only SNPs with a read depth of >50× and genotyped in all individuals were included. Color depicts the genotype at a specific SNP locus (columns), with white and black designating homozygosity, and gray indicating heterozygosity. Each row represents a single individual of *M. guttatus* (rows 1–4; gut‐1 to gut‐4), *M. luteus* (rows 5–8; lut‐1 to lut‐4), *M. peregrinus* (rows 9–12; per‐1 to per‐4), or *M. x robertsii* (rows 13–16; rob‐1 to rob‐4). The four taxa are separated by thick horizontal lines. The x‐axis indicates the relative order of SNPs along linkage groups. Notice the very high level of fixed heterozygosity in the SNP genotypes of *M. x robertsii* and *M. peregrinus*.

Due to the bias in our quantitative measures of allelic dosage, we focused our analysis of genome evolution during hybridization and polyploidization on clear cases of allelic loss (i.e., when one of the parental subgenomes was completely lost). We used the 889 sites (see above) where *M. guttatus* and *M. luteus* were fixed for different alleles in our samples (Fig. 3). For these sites, we found 110 were homozygous in at least one of the four individuals of *M. x robertsii*, and 87 were homozygous in at least one of the four *M. peregrinus* individuals (Fig. 3; Table S1). These sites were distributed in 54 (*M. x robertsii*) and 45 (*M. peregrinus*) probed regions on all the major linkage groups, except LG12 (Table S1). The majority of these sites were homozygous for the *luteus*‐like allele, that is, they were missing the *guttatus*‐like allele (something unlikely to be due to hybridization bias). In *M. x robertsii*, only one site (in LG5) was homozygous for the *guttatus*‐like allele, whereas 109 (in 53 probed regions in 13 LGs) were homozygous for the *luteus*‐like allele (Table S1). In *M. peregrinus*, three sites in two probes (in LG2 and LG14) were homozygous for the *guttatus*‐like allele, and 84 sites in 43 probes (in 13 LGs) were homozygous for the *luteus*‐like allele. The number and identity of these homozygous loci varied among individuals within taxon, with each individual having between 32 and 55 homozygous sites (in 15–29 probes; 7–11 LGs) in *M. x robertsii*, and between 48 and 57 sites (in 25–30 probes; 10–11 LGs) in *M. peregrinus* (Table S1). Therefore, from the 881 informative SNP sites we identified across the genome, between 3.6 and 6.5% are missing one of the expected parental alleles in any one individual of *M. x robertsii* or *M. peregrinus*.

### ALLELE GAIN AND LOSS BETWEEN ***M. X ROBERTSII* AND *M. PEREGRINUS***

By comparing allelic composition between *M. x robertsii* and *M. peregrinus*, we were able to search for changes in read frequency that could reflect gain and loss of individual alleles as well as entire sections of the subgenomes. For instance, if *M. x robertsii* is homozygous at a given SNP site, the presence of a heterozygote at that SNP site in *M. peregrinus* could indicate an allele gain in any one of the duplicated chromosomes. Similarly, a heterozygous SNP site in *M. x robertsii* that is observed as homozygous in *M. peregrinus* could represent a case of allele loss in the allopolyploid. Our search for allelic variants in any *M. peregrinus* individual that were not present in any of the four *M. x robertsii* analyzed here uncovered nine such loci (0.119%, 9/7535 sites; Table S2). We then made this comparison between locally paired taxa of *M. peregrinus* and *M. robertsii*. In the Orkney pair (rob‐1 and per‐3), we found 25 loci with alleles in *M. peregrinus* that were absent in the local *M. x robertsii* (out of 10,612 potentially informative loci). The average frequency of the putatively new alleles in *M. peregrinus* was 0.229 ± 0.125 (mean ± SD). In a similar comparison in Leadhills (rob‐2 and per‐1), we found 21 loci in *M. peregrinus* with an allele not seen in the corresponding *M. x robertsii*. The average frequency of the putatively new alleles was 0.186 ± 0.019. The values for the comparison for a second LED pair (rob‐2 and per‐2) were 16 loci with putatively new alleles (out of 10,611), with an average frequency of the alleles of 0.182 ± 0.013.

**Figure 4:**
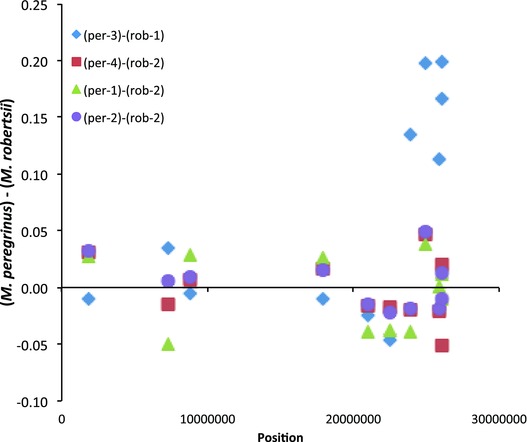
Pairwise difference in allele frequency between individuals of *M. peregrinus* (per‐1 to per‐4) and their closest *M. x robertsii* relative (rob‐1 or rob‐2) at linkage group 14 (LG14). The pairwise allele frequency difference was calculated using a 10 SNP moving average. The horizontal dashed line indicates no allele frequency difference. The x‐axis denotes the position of the informative SNP loci along LG14 in number of base pairs. The individual of *M. peregrinus* from Orkney (per‐3) shows a large allele frequency difference toward one end of LG14.

The analysis of allele absence in *M. peregrinus* compared to *M. x robertsii* revealed that 40 of the 6434 sites that were heterozygous in all individuals of *M. x robertsii*, were observed as homozygotes (0.1 < *q* > 0.9) in at least one individual of *M. peregrinus* (Table S2). The majority of these sites (24/40) were located in a single linkage group (LG14) of the *M. peregrinus* individual from Orkney (per‐3), and these sites were not shared with the other three allopolyploid individuals (Fig. 4; Table S2). The remaining 18 homozygous sites were observed across *M. peregrinus* individuals and linkage groups (Table S2).

## Discussion

### **PARENTAGE OF *M. PEREGRINUS***

Our study represents the first analysis of genome composition in the polyploid Scottish monkeyflower, *M. peregrinus*. Across genome‐wide loci, we found *M. peregrinus* genomes to be highly similar to the genomes of individuals of the widespread sterile triploid hybrid *M. x robertsii*. Almost all the alleles present in *M. peregrinus* and *M. x robertsii* were found in our sampled individuals of the diploid species *M. guttatus* and the tetraploid species *M. luteus*. We found higher levels of heterozygosity per base genotyped in *M. peregrinus* and *M. x robertsii* than in *M. guttatus* and *M. luteus*. For those loci that were fixed for different alleles in *M. guttatus* and *M. luteus* parent, we found that 94.3% showed fixed heterozygosity in *M. peregrinus* and 94.9% in *M. x robertsii* (Fig. 3). Thus, our results corroborate the origin of *M. peregrinus* via whole genome duplication of *M. x* robertsii, itself derived via hybridization from *M. guttatus* and the tetraploid species *M. luteus*. Given that *M. guttatus* and *M. luteus* were introduced into the United Kingdom from the American continent in the 19th century (Roberts 1964; Vallejo‐Marín 2012; Vallejo‐Marín and Lye 2013), *M. peregrinus* is one of a small number of known species (see Introduction) that have arisen via allopolyploid speciation in the last 200 years, and provides the first such study system in the order Lamiales.

Although the triploid *M. x robertsii* suffers from acute sexual sterility (Roberts 1964; Parker 1975), it is not an evolutionary dead‐end. Its ability to propagate via clonal reproduction has allowed this taxon to become part of the ecological landscape of riparian habitats in the United Kingdom (Vallejo‐Marín and Lye 2013), and from it the fertile allopolyploid *M. peregrinus* has emerged. Triploid taxa may give rise to polyploids through a variety of mechanisms including somatic doubling or via mating of unreduced gametes (Mable 2003; Mason and Pires 2015). Although these are improbable events, the ecological success of the triploid hybrid *M. x robertsii* as clonal populations seems to have allowed this probabilistic barrier to have been overcome, and a new allopolyploid to have formed. Other recently formed allopolyploids seem to have emerged from a similar route: for example, the allohexaploid *S. cambrensis* emerged from triploid hybrid, *S. x baxteri* whose parental species are *S. vulgaris* (tetraploid) and *S. squalidus* (diploid; Hegarty et al. 2005). The allopolyploid *S. anglica* (2*n* = 120–124) formed in around 1890 from *S. x townsendii* which is a sterile hybrid of *S. maritima* (2*n* = 60) and *S. alterniflora* (2*n* = 62; Baumel et al. 2002).

### MULTIPLE ORIGINS OF THE ALLOPOLYPLOID

Our data show that *M. peregrinus* has formed at least twice, independently, from local populations of *M. x robertsii*, both on the Scottish mainland and on the Orkney Islands. Populations of *M. peregrinus* are very similar in the genic regions that we sequenced to their local populations of *M. x robertsii*, providing strong evidence for local origins from nearby populations of the sterile hybrid. The large distance between the two areas currently known to contain *M. peregrinus*, including 16 km of ocean, make a very strong cumulative case for the independent origins of this allopolyploid. Multiple origins of allopolyploids have been found in other recently formed allopolyploid species (Soltis and Soltis 1999; Soltis et al. 2009) including *T. mirus* and *T. miscellus* (Symonds et al. 2010) and *S. cambrensis* (Ashton and Abbott 1992).

It is likely that *M. peregrinus* is underreported in the field as many of the diagnostic characteristics that differentiate it from other *Mimulus* taxa in the United Kingdom, such as pollen and stomata size (Vallejo‐Marín 2012), can only be assessed in laboratory settings. Moreover, the presence of viable pollen and floral characteristics that are superficially similar to some forms of *M. guttatus*, represent a challenge to recognize *M. peregrinus* as a hybrid taxon (Silverside 1998; Stace 2010). The widespread distribution of its parental taxon, *M. x robertsii*, throughout the British Isles (Preston et al. 2002) may provide ample opportunities for additional origins of *M. peregrinus*, but this remains to be established in future field surveys.

### **POLYPLOIDIZATION IN *MIMULUS***

Chromosomal change, including aneuploidy and polyploidization, is considered to be an important mechanism of speciation in many plant groups (Otto and Whitton 2000; Levin 2002), including *Mimulus* (Vickery 1995). In *Mimulus*, phylogenetic analyses have revealed at least 13 polyploidization and 15 aneuploidization events within western North American species (Beardsley et al. 2004). However, genetic evidence in support of this view is only recently beginning to accumulate. For example, genetic analyses of three chloroplast and six nuclear loci revealed that the North American allotetraploid *M. sookensis* (2*n* = 4x = 56), evolved via hybridization and genome duplication between two diploid taxa, *M. guttatus* and *M. nasutus* (Benedict et al. 2012). As in *M. peregrinus*, genetic analysis show elevated heterozygosity and multiple origins for *M. sookensis* (11 origins in this case), but unlike the case of *M. x robertsii* and *M. peregrinus*, triploid hybrids between *M. guttatus* and *M. nasutus*, expected if tetraploids arise via a triploid bridge stage (Ramsey and Schemske 1998; Husband 2004), are not known from nature (Modliszewski and Willis 2012). Importantly, experimental crosses indicate a lack of reproductive barriers between populations of *M. sookensis* stressing the potential for multiple origins to contribute to genetic diversity of nascent polyploids (Modliszewski and Willis 2012; Soltis et al. 2014). The fact that *M. sookensis* and *M. peregrinus* share a parent in common provides an exciting opportunity to compare closely related allopolyploid taxa of different ages, and which have arisen via hybridization between *M. guttatus* and taxa with similar (*M. nasutus*) or different (*M. luteus*) chromosome numbers (Buggs 2012).

#### Fixed heterozygosity in M. luteus

*Mimulus luteus*, one of the parents of *M. peregrinus*, is tetraploid (Mukherjee and Vickery 1962). Our findings of very high levels of heterozygosity in *M. luteus*, including in the two advanced generation inbred lines studied here (lut‐2 and lut‐4), indicate that heterozygosity is fixed at many loci in *M. luteus* (Fig. S2), supporting a previous hypothesis that this species is itself an allopolyploid (Mukherjee and Vickery 1962). Further studies are needed to establish the parentage and age of this allopolyploid, and in particular, whether *M. guttatus* is one of its parental species.

### GENOMIC COMPOSITION OF A YOUNG ALLOPOLYPLOID

We found that overall allele dosage ratios within *M. peregrinus* and *M. x robertsii* at loci that have alternatively fixed alleles in *M. guttatus* and *M. luteus* departed slightly from the expected genomic composition of a 1:2 ratio within a diploid × tetraploid hybrid. Overall, a bias was found in favor of *M. guttatus* alleles. This discrepancy is partly due to hybridization bias in favor of alleles found in the reference genome used to design the probes, which mainly favored *M. guttatus* alleles. Using alternative approaches such as whole genome sequencing or RADseq (Buggs et al. 2012b) could reduce biases against sequencing divergent copies of duplicated loci by bypassing the hybridization step used in sequence capture. In addition, future analyses of the *M. luteus* genome could indicate the extent to which *M. luteus* has diploidized at some loci (diploidization defined here as the process where duplicated gene copies are lost, Conant et al. 2014). The absence of one of the homeologous loci, for example, due to deletion, in *M. luteus* could result in an increase in the observed read depth of *M. guttatus* alleles in their hybrid offspring. In this regard, elucidating the origin and genomic structure of *M. luteus* will help refine the hypotheses for the expected genomic composition of *M. peregrinus*.

The allele dosage ratio analysis further uncovered a relatively large number of loci that were missing one of the expected parental alleles (Fig. 2, Table S1), which could represent either candidates for allele loss in *M. peregrinus* or unsampled genetic variation in the parental taxa. The confounding effect of unsampled variation in the current analysis may be particularly acute in the high incidence of “subgenome loss” observed in both *M. x robertsii* and *M. peregrinus* (Fig. 1) relative to *M. guttatus* and *M. luteus*. We found more than 100 loci in which the *M. guttatus* allele appears to be missing. It is possible that the diploid subgenome (*M. guttatus*) is more likely to be lost during hybridization than the tetraploid subgenome (*M. luteus*). However, a more likely explanation is simply that our limited sample of *M. guttatus* individuals did not capture some of the variation segregating within this species, and thus that the alleles used to depict *luteus*‐specific reads were also present in (unsampled) *M. guttatus*. Evidence in support of the hypothesis that unsampled variation could create the pattern of deletions observed here, includes that no probed regions showed a complete loss of one of the parental copies—only individual SNPs or subsets of SNPs within a given probe showed signs of deletion—and that most loci with missing alleles were observed in both *M. peregrinus* and *M. x robertsii* (Fig. 3), implying that missing alleles were not exclusively associated with genome duplication.

However, our data on allelic presence/absence in the specific comparison between the genomes of *M. x robertsii* and *M. peregrinus* raise the possibility that a small level of genome evolution is occurring in neoallopolyploids. In this comparison, we found evidence consistent with either gain (10/9362 sites) or loss/conversion (42/6625) of alleles following polyploidization (Table S2). In contrast to the allele dosage ratio analysis, these changes must have occurred principally during or after whole genome duplication. The evidence for genome evolution in *M. peregrinus* is particularly compelling in the pattern of allele loss/conversion observed in linkage group 14 (LG14) in the individual from the Orkney Islands (per‐3; Fig. 4). The pairwise comparison of allele frequencies between per‐3 and the closest *M. x robertsii* sample (rob‐1) show a consistent pattern of differences in allele frequencies in 24 SNP loci across seven different probes in the terminal portion of LG14, in a region spanning approximately 2,500,000 bp. (Fig. 4, Table S2). The average allele frequency difference between per‐3 and rob‐1 is 0.18 ± 0.05 (mean ± SD), which is very close to the expected change in frequency caused by the loss/conversion of a single allele in a hexaploid (1/6 = 0.167). Although we cannot completely rule out that some of this “gain” and “loss/conversion” of alleles represents unsampled genetic variation in the exact parents of the studied polyploids, the assumption that the studied individuals of *M. x robertsii* reflect the genomic composition of the direct ancestors of *M. peregrinus*, seems reasonable given that we included in our analyses the local *M. x robertsii* for the two origins of *M. peregrinus* (Fig. 1).

Ultimately, studying synthetic polyploids may provide a more direct estimation of genome restructuring following whole genome duplication (Hegarty et al. 2013). For example, a study of synthetic individuals of *M. sookensis* found 10 instances of fragment loss among 48 individuals from a polyploid backcross (BC1N) between *M. guttatus* and *M. nasutus* genotyped at seven fragment‐length polymorphism markers (Modliszewski and Willis 2014). This number of fragment‐loss events is lower than the expectation if loss occurs via homeologous recombination (Modliszewski and Willis 2014), but highlights the potential of genome restructuring to occur relatively rapidly. In synthetic *M. sookensis*, there is little evidence that this level of fragment loss results in phenotypic variation among synthetic polyploids, but our understanding of the link between genome restructuring in polyploids and phenotypic variation is still in its infancy.

In tetraploid species of *Tragopogon*, which have arisen via allopolyploidization of two diploid species within the last century, substantial losses of parental alleles have been found (Buggs et al. 2009, 2012a; Tate et al. 2009; Soltis et al. 2012). Evidence from cytogenetic studies strongly suggests that some of these parental alleles have been removed from the genome via nonhomeologous recombination (Chester et al. 2012, 2013, 2014). However, not all documented cases of parent allele loss can be accounted for in this way, suggesting that smaller scale genomic rearrangement or gene conversion events may also play a role (Chester et al. 2012; Buggs et al. 2012aa). In contrast, other allopolyploid alleles appear to have much more stable genomes (Liu et al. 2001; Mandáková et al. 2014). In the long term, we do not know if genomic changes in allopolyploids provide a source of variation that may contribute to their success, or whether genomic variants tend to be of low fitness and only individuals with stable genomes ultimately survive (Soltis et al. 2010).

It is therefore hard to predict the long‐term evolutionary trajectory of *M. peregrinus*. On the one hand, each population currently has limited genetic diversity, which as with all neoallopolyploids may represent a serious challenge to the ability of these taxa to respond to changing environments (Levin 2002). On the other hand, its multiple origins and apparent rapid genome evolution could provide the variation that it needs to become a successful species (Modliszewski and Willis 2012; Soltis et al. 2014). Whether it will remain a short‐lived scientific curiosity like the allotetraploid *Senecio eboracensis* (Lowe and Abbott 2004), which is now extinct in the wild, or spread well beyond its place of origin like allopolyploid *S. anglica* (Ainouche et al. 2009) remains to be seen.

## Supporting information


**Figure S1**. Positions of probes used in the sequence capture experiment mapped on the 14 major linkage groups (scaffolds) of *M. guttatus* (genome version 2.0, www.phytozome.net).
**Figure S2**. Heterozygosity plot of 16 *Mimulus* spp. individuals across 20,749 biallelic SNPs genotyped at a minimum read depth of 50× in all individuals.
**Figure S3**. Neighbor joining tree of 16 *Mimulus* spp. showing bootstrap support for all nodes.
**Figure S4**. Allele frequency for 881 SNPs in four individuals of *M. x robertsii* (A) and four of *M. peregrinus* (B) mapped against the 14 major linkage groups of the *M. guttatus* reference genome.
**Table S1**. List of SNP loci showing a departure from expected heterozygosity in *M. x robertsii* and *M. peregrinus* based on expectation from parental genotypes.
**Table S2**. Location and identity of the SNP sites in which a loss or gain of an allele was detected between *M. x robertsii* and *M. peregrinus*.
Additional Supplementary Material: Bioinformatic commands for alignment and SNP genotyping.
