# Supplementary material for: Speciation by genome duplication: Repeated origins and genomic composition of the recently formed allopolyploid species Mimulus peregrinus
Source: Evolution. 2015 May 27;69(6):1487–500. doi: 10.1111/evo.12678 (PMC5033005; doi:10.1111/evo.12678)
Supplement: Supplementary file 1 — Figure S1. Positions of probes used in the sequence capture experiment mapped on the 14 major linkage groups (scaffolds) of M. guttatus (genome version 2.0, www.phytozome.net). Figure S2. Heterozygosity plot of 16 Mimulus spp. individuals across 20,749 biallelic SNPs genotyped at a minimum read depth of 50× in all individuals. Figure S3. Neighbor joining tree of 16 Mimulus spp. showing bootstrap support for all nodes. Figure S4. Allele frequency for 881 SNPs in four individuals of M. x robertsii (A) and four of M. peregrinus (B) mapped against the 14 major linkage groups of the M. guttatus reference genome. Table S1. List of SNP loci showing a departure from expected heterozygosity in M. x robertsii and M. peregrinus based on expectation from parental genotypes. Table S2. Location and identity of the SNP sites in which a loss or gain of an allele was detected between M. x robertsii and M. peregrinus. Additional Supplementary Material: Bioinformatic commands for alignment and SNP genotyping. [file EVO-69-1487-s001.zip › evo12678-sup-0001-SupMat/evo12678-sup-0005-Text.docx]

# Speciation by genome duplication: Repeated origins and genomic composition of the recently formed allopolyploid species *Mimulus peregrinus*

Mario Vallejo-Marín^1,^*

Richard J. A. Buggs^2^

Arielle M. Cooley^3^

Joshua R. Puzey^4^

^1^ Biological and Environmental Sciences, University of Stirling, Stirling, United Kingdom. FK9 4LA.

* Author for correspondence (mario.vallejo@stir.ac.uk)

^2^ School of Biological and Chemical Sciences, Queen Mary University of London, London, United Kingdom. E1 4NS.

^3^ Biology Department, Whitman College. Walla Walla, Washington, United States of America. 99362.

^4^ Department of Biology, College of William and Mary, Williamsburg, Virginia, United States of America. 23185.

# Bioinformatic commands

Alignment of short reads to *M. guttatus* genome:

bowtie2 --local --fast-local -x Mguttatus_v2.0 -1 fastq-1 -2 fastq-2 -S out.sam

Genotype calling with GATK Unified Genotyper:

java -Xmx5g -jar GenomeAnalysisTK.jar -R Mguttatus_v2.0.fa -T UnifiedGenotyper -rf MaxInsertSize --maxInsertSize 10000 -rf DuplicateRead -rf BadMate -rf BadCigar --min_base_quality_score 25 -rf MappingQuality --min_mapping_quality_score 25 -ploidy 2 --genotype_likelihoods_model BOTH --output_mode EMIT_ALL_SITES --max_alternate_alleles 2 --standard_min_confidence_threshold_for_emitting 30 -dcov 500 -I BAMs -o out.vcf

GATK in polyploidy mode was used to identify and remove indels and sites with two or more alternate alleles:

java -Xmx5g - GenomeAnalysisTK.jar -R Mguttatus_v2.0.fa -T UnifiedGenotyper -rf MaxInsertSize --maxInsertSize 10000 -rf DuplicateRead -rf BadMate -rf BadCigar --min_base_quality_score 25 -rf MappingQuality --min_mapping_quality_score 25 -ploidy 4 --genotype_likelihoods_model BOTH --output_mode EMIT_ALL_SITES --max_alternate_alleles 4 --standard_min_confidence_threshold_for_emitting 30 -dcov 500 –I BAMS –o out.vcf
