# Supplementary figures and images for: Speciation by genome duplication: Repeated origins and genomic composition of the recently formed allopolyploid species Mimulus peregrinus
Source: Evolution. 2015 May 27;69(6):1487–500. doi: 10.1111/evo.12678 (PMC5033005; doi:10.1111/evo.12678)

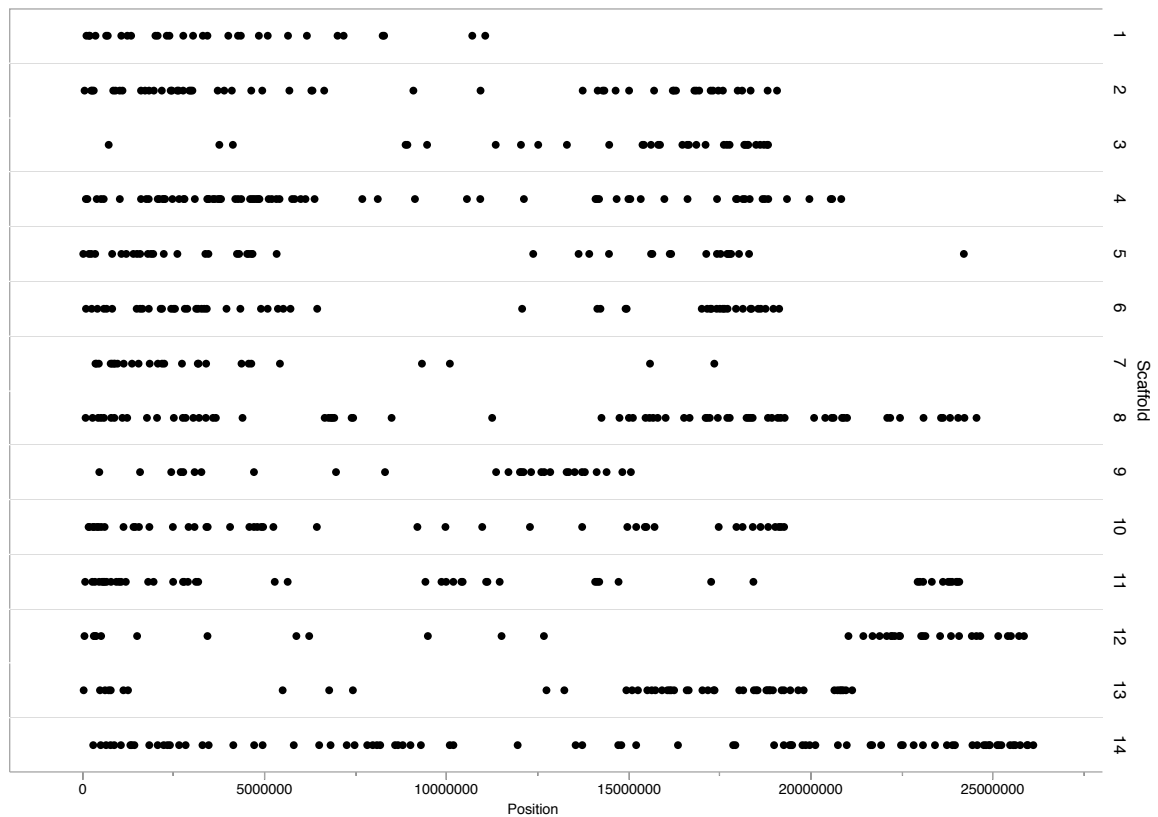

**Figure S1. Positions of probes on 14 major linkage groups** (uniquely mapped probes on v.2 genome).

Supplement: Supplementary file 1 — Figure S1. Positions of probes used in the sequence capture experiment mapped on the 14 major linkage groups (scaffolds) of M. guttatus (genome version 2.0, www.phytozome.net). Figure S2. Heterozygosity plot of 16 Mimulus spp. individuals across 20,749 biallelic SNPs genotyped at a minimum read depth of 50× in all individuals. Figure S3. Neighbor joining tree of 16 Mimulus spp. showing bootstrap support for all nodes. Figure S4. Allele frequency for 881 SNPs in four individuals of M. x robertsii (A) and four of M. peregrinus (B) mapped against the 14 major linkage groups of the M. guttatus reference genome. Table S1. List of SNP loci showing a departure from expected heterozygosity in M. x robertsii and M. peregrinus based on expectation from parental genotypes. Table S2. Location and identity of the SNP sites in which a loss or gain of an allele was detected between M. x robertsii and M. peregrinus. Additional Supplementary Material: Bioinformatic commands for alignment and SNP genotyping. [file EVO-69-1487-s001.zip › evo12678-sup-0001-SupMat/evo12678-sup-0001-FigureS1.pdf]

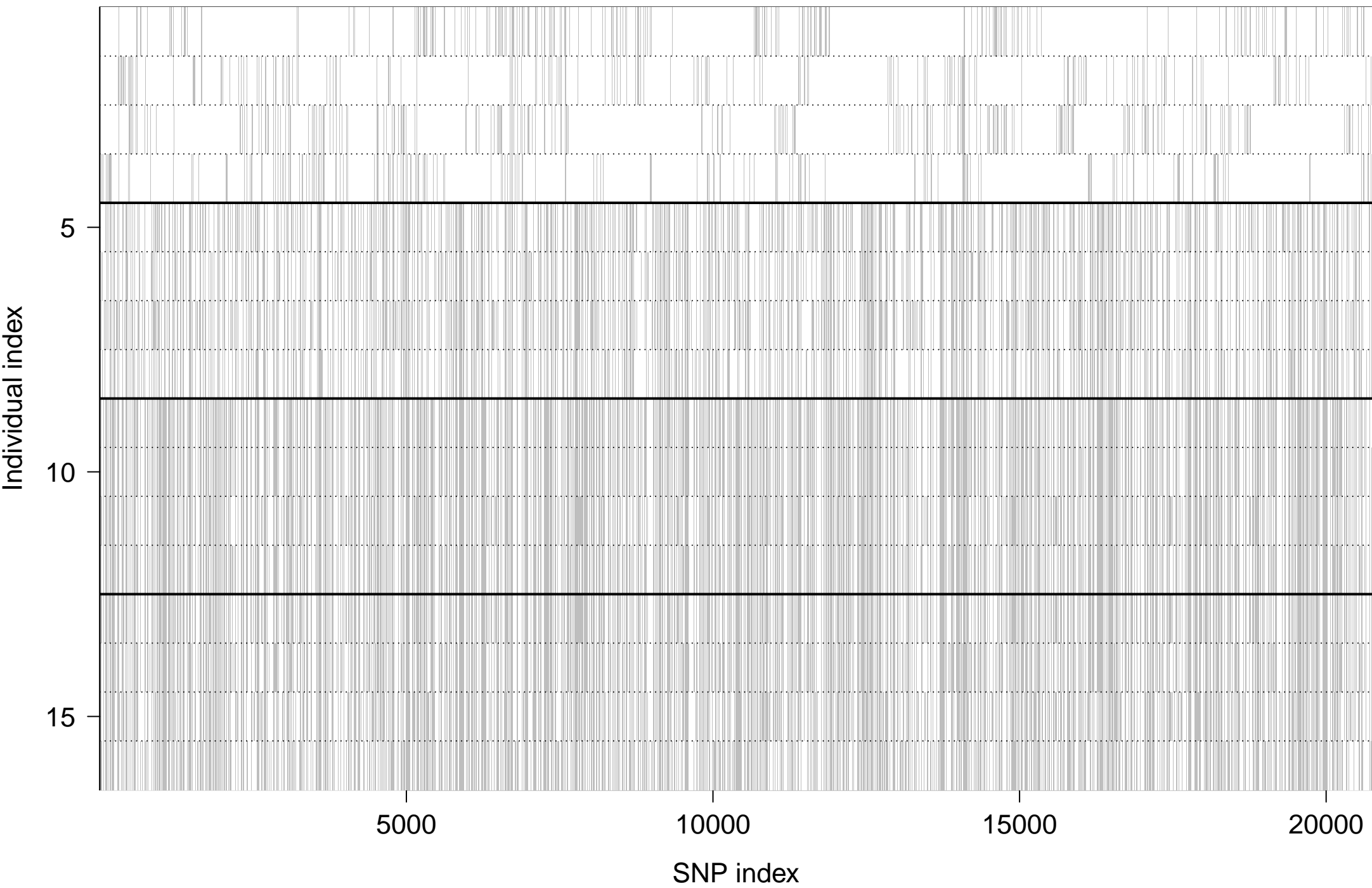

Supplement: Supplementary file 1 — Figure S1. Positions of probes used in the sequence capture experiment mapped on the 14 major linkage groups (scaffolds) of M. guttatus (genome version 2.0, www.phytozome.net). Figure S2. Heterozygosity plot of 16 Mimulus spp. individuals across 20,749 biallelic SNPs genotyped at a minimum read depth of 50× in all individuals. Figure S3. Neighbor joining tree of 16 Mimulus spp. showing bootstrap support for all nodes. Figure S4. Allele frequency for 881 SNPs in four individuals of M. x robertsii (A) and four of M. peregrinus (B) mapped against the 14 major linkage groups of the M. guttatus reference genome. Table S1. List of SNP loci showing a departure from expected heterozygosity in M. x robertsii and M. peregrinus based on expectation from parental genotypes. Table S2. Location and identity of the SNP sites in which a loss or gain of an allele was detected between M. x robertsii and M. peregrinus. Additional Supplementary Material: Bioinformatic commands for alignment and SNP genotyping. [file EVO-69-1487-s001.zip › evo12678-sup-0001-SupMat/evo12678-sup-0002-FigureS2.pdf]

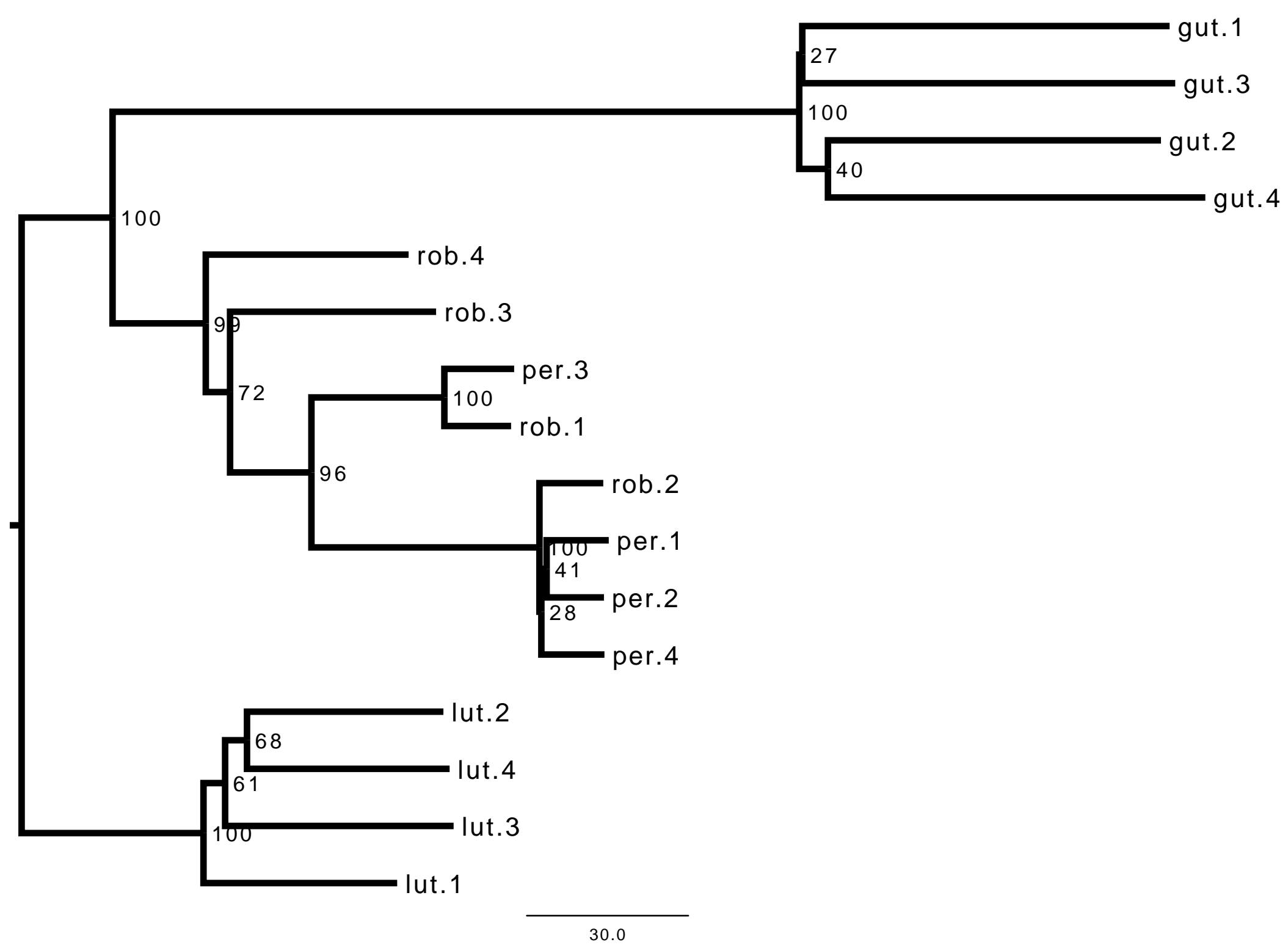

Supplement: Supplementary file 1 — Figure S1. Positions of probes used in the sequence capture experiment mapped on the 14 major linkage groups (scaffolds) of M. guttatus (genome version 2.0, www.phytozome.net). Figure S2. Heterozygosity plot of 16 Mimulus spp. individuals across 20,749 biallelic SNPs genotyped at a minimum read depth of 50× in all individuals. Figure S3. Neighbor joining tree of 16 Mimulus spp. showing bootstrap support for all nodes. Figure S4. Allele frequency for 881 SNPs in four individuals of M. x robertsii (A) and four of M. peregrinus (B) mapped against the 14 major linkage groups of the M. guttatus reference genome. Table S1. List of SNP loci showing a departure from expected heterozygosity in M. x robertsii and M. peregrinus based on expectation from parental genotypes. Table S2. Location and identity of the SNP sites in which a loss or gain of an allele was detected between M. x robertsii and M. peregrinus. Additional Supplementary Material: Bioinformatic commands for alignment and SNP genotyping. [file EVO-69-1487-s001.zip › evo12678-sup-0001-SupMat/evo12678-sup-0003-FigureS3.pdf]

A

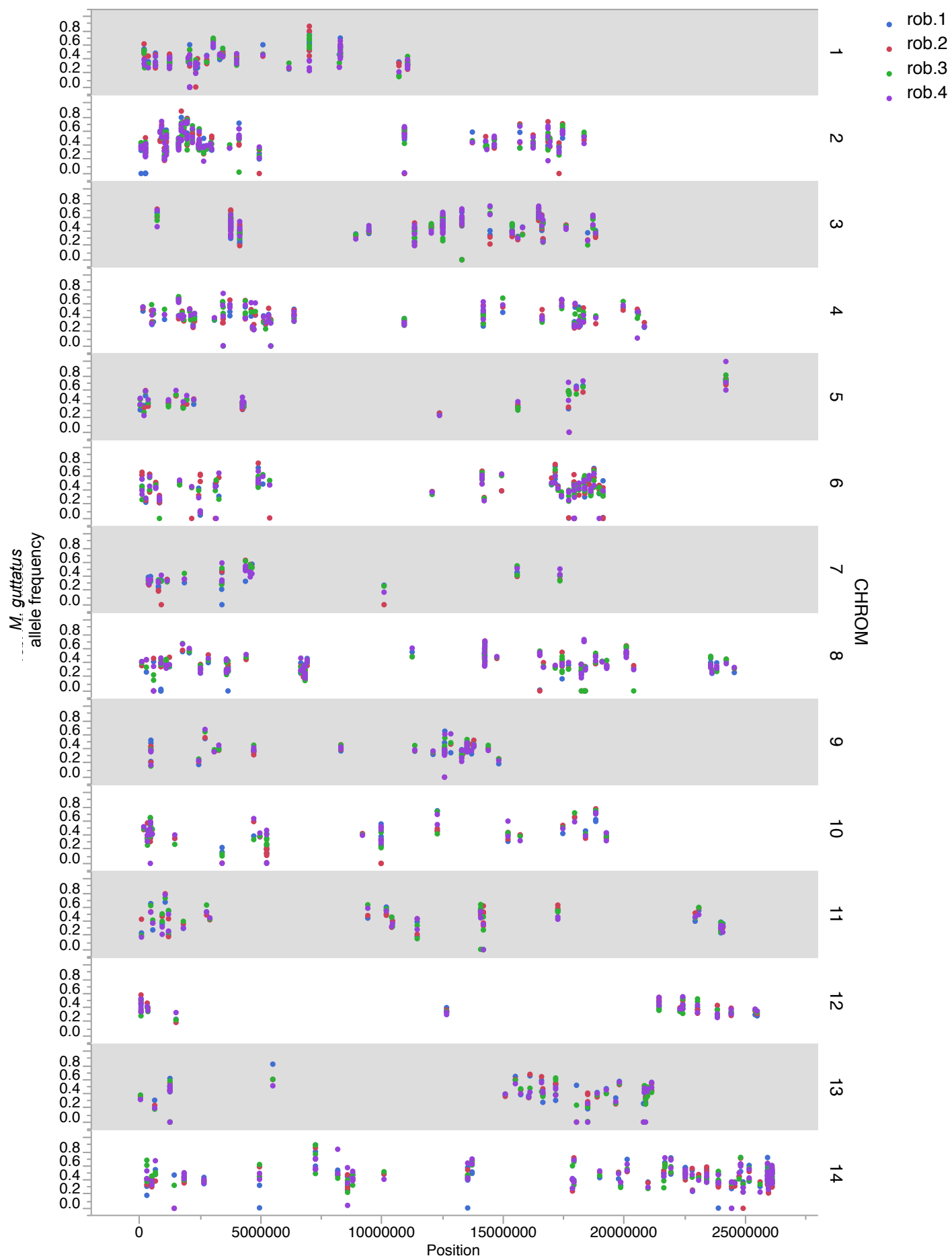

B

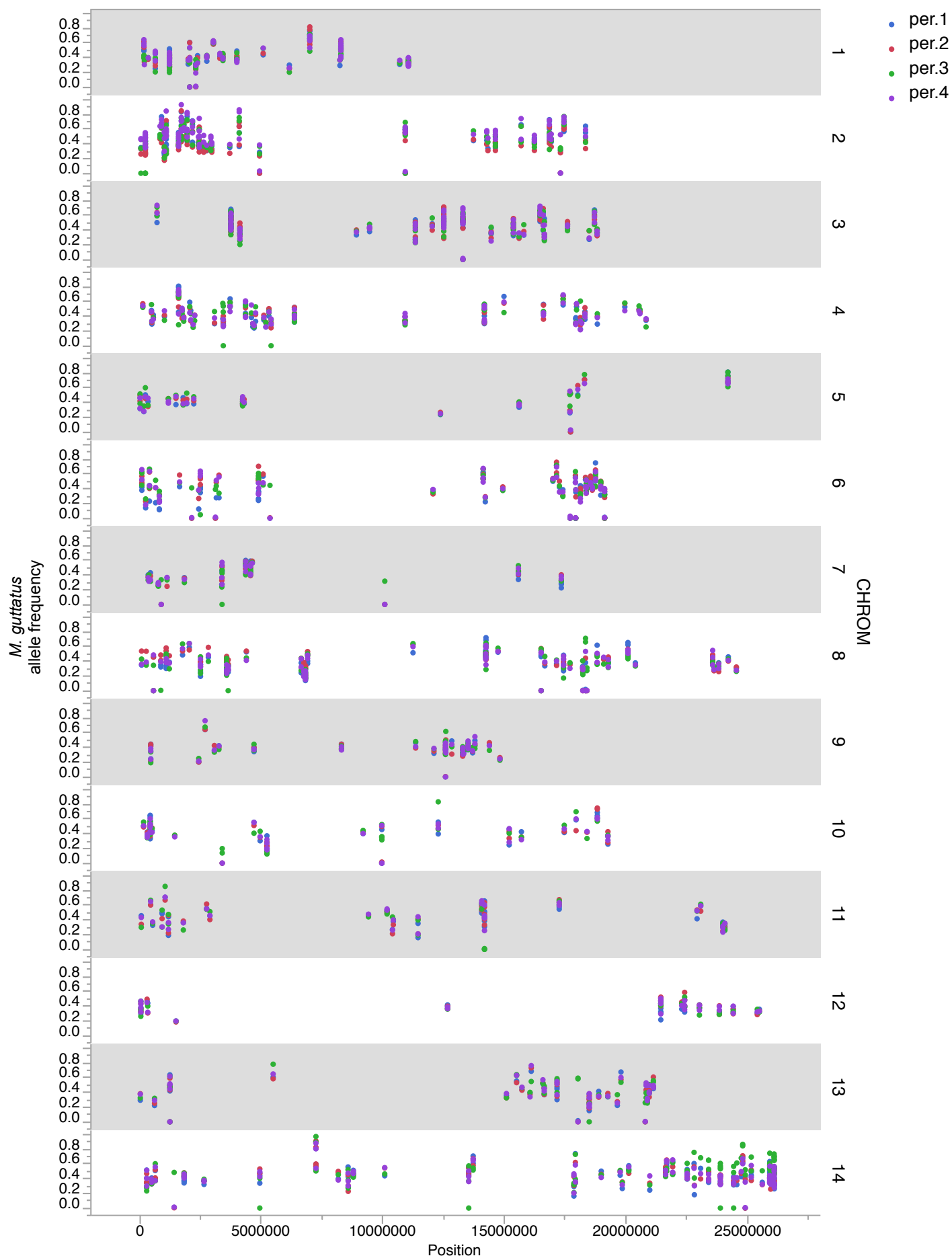

Supplement: Supplementary file 1 — Figure S1. Positions of probes used in the sequence capture experiment mapped on the 14 major linkage groups (scaffolds) of M. guttatus (genome version 2.0, www.phytozome.net). Figure S2. Heterozygosity plot of 16 Mimulus spp. individuals across 20,749 biallelic SNPs genotyped at a minimum read depth of 50× in all individuals. Figure S3. Neighbor joining tree of 16 Mimulus spp. showing bootstrap support for all nodes. Figure S4. Allele frequency for 881 SNPs in four individuals of M. x robertsii (A) and four of M. peregrinus (B) mapped against the 14 major linkage groups of the M. guttatus reference genome. Table S1. List of SNP loci showing a departure from expected heterozygosity in M. x robertsii and M. peregrinus based on expectation from parental genotypes. Table S2. Location and identity of the SNP sites in which a loss or gain of an allele was detected between M. x robertsii and M. peregrinus. Additional Supplementary Material: Bioinformatic commands for alignment and SNP genotyping. [file EVO-69-1487-s001.zip › evo12678-sup-0001-SupMat/evo12678-sup-0004-FigureS4.pdf]
